# Management of rasopathies

**DOI:** 10.1186/1687-9856-2013-S1-O7

**Published:** 2013-10-03

**Authors:** Han-Wook Yoo

**Affiliations:** 1 Department of Pediatrics, Medical Genetics Center, Asan Medical Center Children’s Hospital, University of Ulsan College of Medicine, Seoul, Korea

## 

Noonan syndrome (NS) and NS-related disorders (Cardio-Facio-Cutaneous (CFC) syndrome, Costello syndrome, LEOPARD (Lentigines, ECG conduction abnormalities, Ocular hypertelorism, Pulmonic stenosis, Abnormal genitalia, Retardation of growth and sensory neural Deafness) syndrome) share common clinical features characterized by unique facial features, postnatal growth failure, psychomotor retardation, ectodermal abnormalities, congenital heart diseases, chest & skeletal deformity and delayed puberty. During last decade, strident progress has been made in molecular understanding of NS. The functional alterations of the Ras-mitogen-activated protein kinase (MAPK) pathway are caused by the mutation in more than 10 genes (*PTPN11*, *SOS1*, *RAF1*, *SHOC2*, *BRAF*, *KRAS*, *NRAS*, *HRAS*, *MEK1*, *MEK2*). Thus, NS and NS-related disorders are called RASopathies as a disease group. *PTPN11* (40-50%), *SOS1* (10%–20%), and *RAF1* (3%-17%) mutations are common in NS patients. Noonan syndrome and its related disorders are not rare as a whole. Since their disease natural course and management are different, it is important to recognize RASopathies and differentiate them primarily based on typical clinical feature. By utilizing DNA testing, the confirmatory diagnosis can be made. Multi-systemic involvement in RASopathies requires multidisciplinary evaluation and regular monitoring for each special clinical issue. It involves whole spectra of clinical issues of cardiovascular, growth & endocrine, neuro-cognitive, developmental, skeletal & orthopedic, opthalmo-otolaryngological, GI-nutritional, dental, hemato-oncological and ectodermal systems. For instances, surgical intervention is required for congenital heart defects and cryptoorchidism. Before the surgery, bleeding diathesis should be excluded. Also the risk for malignant hyperthermia has to be considered in choosing anesthetics. Special education might be required in 10-40% of NS patients. However, NS patients carrying the mutation in the *SOS1* gene and N380D or N380S mutation in the *PTPN11* gene tend to show normal cognitive function. Many NS infants have feeding difficulties with poor suck and prolonged feeding time and may require tube feeding in 24% of NS infants. Most NS patients show normal levels of IGF-1 and IGF-BP3, indicating growth hormone (GH) deficiency is not culpable for postnatal growth failure. However, some studies have demonstrated subnormal overnight mean growth hormone concentration, suggestive of impaired GH secretion. The rhGH therapy in NS has been reported to be effective to improve both the height velocity and the final adult height. In this review, the constellation of overlapping clinical features of RASopathies will be described based on genotype as well as their differential diagnostic points and management.

